# Fibroblast-Conditioned Media Enhance the Yield of Microglia Isolated from Mixed Glial Cultures

**DOI:** 10.1007/s10571-022-01193-9

**Published:** 2022-02-12

**Authors:** Jian Hu, Peng Wang, Zhengyi Wang, Yuyun Xu, Wenshuo Peng, Xiongjian Chen, Yani Fang, Liyun Zhu, Dongxue Wang, Xue Wang, Li Lin, Lixin Ruan

**Affiliations:** 1 Pingyang Affiliated Hospital of Wenzhou Medical University, No. 555 Kunao Dadao, Kunyang Town, Wenzhou, 325400 Zhejiang China; 2 School of Pharmaceutical Sciences, Wenzhou Medical University, Chashan Higher Education Park, Wenzhou, 325035 Zhejiang China; 3 The First Affiliated Hospital of Wenzhou Medical University, Wenzhou, 325000 Zhejiang China

**Keywords:** Primary microglia, Mild trypsinization, Fibroblast-conditioned media, Phenotype, Immune response

## Abstract

Microglia are the main immune cells of the central nervous system (CNS) and comprise various model systems used to investigate inflammatory mechanisms in CNS disorders. Currently, shaking and mild trypsinization are widely used microglial culture methods; however, the problems with culturing microglia include low yield and a time-consuming process. In this study, we replaced normal culture media (NM) with media containing 25% fibroblast-conditioned media (F-CM) to culture mixed glia and compared microglia obtained by these two methods. We found that F-CM significantly improved the yield and purity of microglia and reduced the total culture time of mixed glia. The microglia obtained from the F-CM group showed longer ramified morphology than those from the NM group, but no difference was observed in cell size. Microglia from the two groups had similar phagocytic function and baseline phenotype markers. Both methods yielded microglia were responsive to various stimuli such as lipopolysaccharide (LPS), interferon-γ (IFN-γ), and interleukin-4 (IL-4). The current results suggest that F-CM affect the growth of primary microglia in mixed glia culture. This method can produce a high yield of primary microglia within a short time and may be a convenient method for researchers to investigate inflammatory mechanisms and some CNS disorders.

## Introduction

Microglia, the resident immune cells of the CNS (Tay et al. 2019; Prinz et al. 2021), play an increasingly important role in maintaining normal brain function (Loane and Kumar 2016; Deczkowska et al. 2018). Microglia can be activated by many stimuli as well as changes in morphology, cytokine expression, gene expression and other functions (Norden et al. 2016). Activated microglia are generally categorized into two subsets including the “classically activated”, pro-inflammatory M1 phenotype and the “alternatively activated” anti-inflammatory M2 phenotype (Michelucci et al. 2009; Parisi et al. 2018; Mesquida-Veny et al. 2021). In vitro, the M1 phenotype can be achieved by stimulating cells with lipopolysaccharides (LPS) or/and interferon-γ (IFN-γ), which can induce microglia to produce pro-inflammatory factors that have the ability to aggravate an initial brain injury and the associated dysfunction (Colton 2009; Pan et al. 2015), while interleukin-4 (IL-4) and IL-13 are typically used to induce an M2-like phenotype (Eggen et al. 2013; Dort et al. 2019). M2 phenotype microglia produce various protective growth factors, including brain-derived neurotrophic factor (BDNF), vascular endothelial growth factor (VEGF), and platelet-derived growth factor (PDGF), that are important for angiogenesis, neurogenesis, and wound healing (Cherry et al. 2014).

Presently, primary microglia isolated from embryonic or neonatal animals are extensively used to explore mechanisms of diseases or drugs that target microglia. The classical protocol to obtain microglia from primary mixed glial cultures consists of shaking (Hassan et al. 1991) and mild trypsinization (Saura et al. 2003). Mild trypsinization has been reported to produce a higher yield and purity. However, microglia obtained by the mild trypsinization method exhibit more of a “resting” state than those produced by shaking, which are more representative of the normal physiological state (Lin et al. 2017).

In vitro, the developmental program of microglia is controlled by various molecules that include but are not limited to transcription factors, growth factors, and chemokines. However, dissection of the meninges from the cortex is necessary to avoid contamination of the final mixed glia culture by meningeal cells and fibroblasts when culturing primary microglia in vivo (Schildge et al. 2013). Fibroblasts, which secrete many cytokines, such as growth factors, affect the growth microenvironment of other cells (Baglole et al. 2006; Oyanagi et al. 2014). Indeed, previous studies have reported that co-culture with fibroblasts affects the survival and function of peripheral cells (Haubner et al. 2015; Jeong et al. 2016a, 2016b; Aberer et al. 2018), while culturing CNS cells with fibroblasts, especially glia, is rarely reported. In this report, microglia were isolated from primary rat mixed glia that cultured with fibroblast-conditioned media (F-CM). We confirmed that F-CM can improve the yield of microglia and shorten the culture time of mixed glial cells compared to normal media (NM). The morphology, gene expression profiles and other characteristics of microglia subjected to the two different treatments were also assessed to explore the effects of F-CM on microglia.

## Materials and Methods

### Reagents and Antibodies

The DMEM/F12, DMEM, 0.25% trypsin–EDTA, 0.25% trypsin and P/S used for cell culture were from obtained from Gibco (Thermo Fisher Scientific, USA). Fetal bovine serum (FBS) was purchased from HyClone (Thermo Fisher Scientific, USA). The primary antibody against Iba-1 (Cat# ctf4377, 1:500, Wakao Osaka, Japan) and the Alexa Fluor 488-conjugated secondary antibody (ab150157, 1:800, abcam) were obtained from Abcam (Cambridge, MA, USA). The TRITC-conjugated secondary antibody was purchased from Bosterbio (Wuhan, China). The CD11b-APC (Cat# 101211, 1:20, Biolegend, San Diego, CA, USA) and CD45-APC antibodies (Cat# 103111, 1:20, Biolegend) used for flow cytometry detection were purchased from BD and eBioscience, respectively. LPS, poly-D-lysine (PDL) and fluorescent beads were obtained from Sigma-Aldrich (St. Louis, MO, USA). Recombinant rat IL-4 and IFN-γ were from purchased R&D system (Minnesota, USA).

### Fibroblast Culture and Conditioned Media Preparation

NIH-3T3 fibroblasts (ATCC) were cultured in DMEM supplemented with 10% FBS and 1% P/S in a 100-mm cell culture dish at 37 °C in a humidified atmosphere of 95% air/5% CO_2_. Cells were rinsed with PBS, and the growth medium was replaced when fibroblast culture became confluent. After 24 h, the medium was collected and centrifuged at 3000 × rcf for 10 min to remove cell debris.

### Primary Rat Microglia Culture

**Fig. 1:**
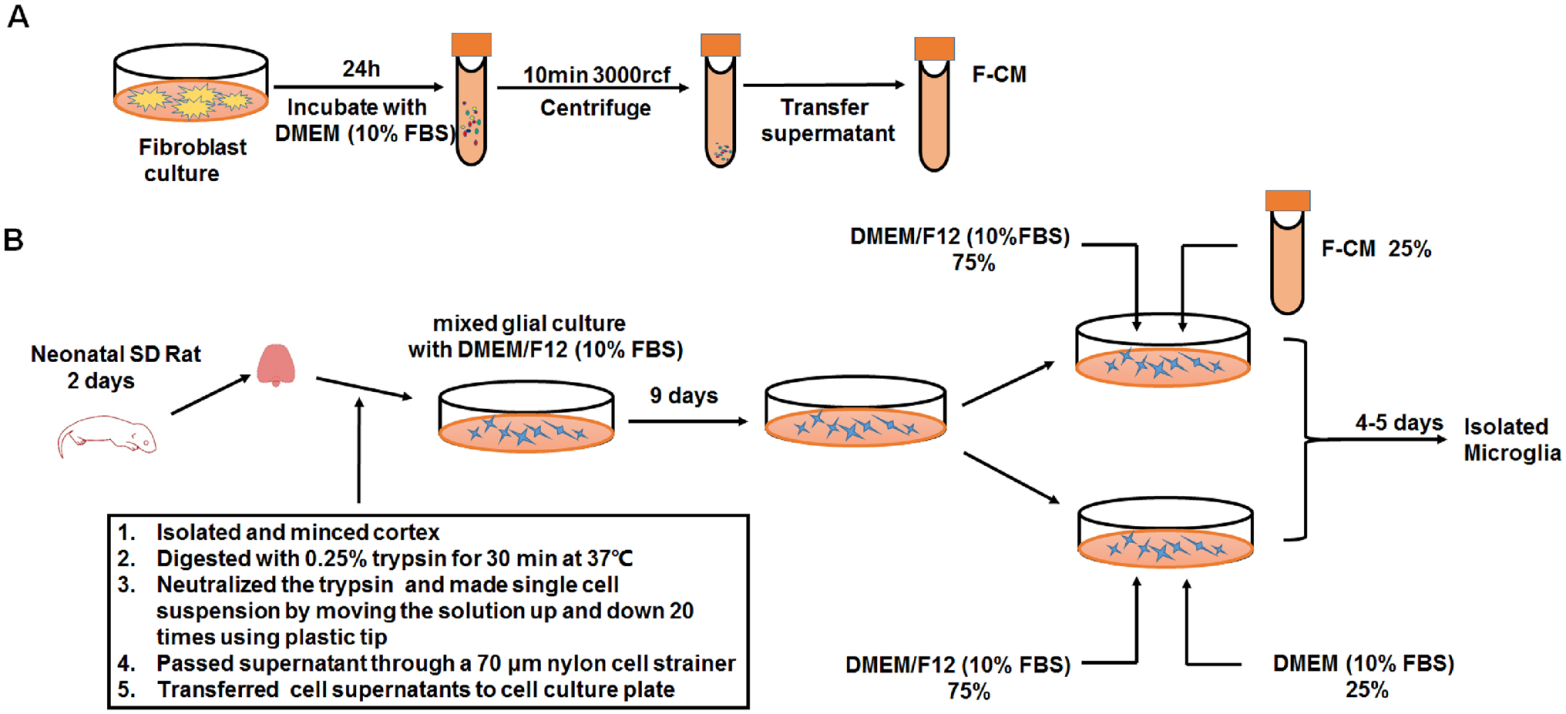
Procedure for preparation of F-CM and culturing cells. **a** Fibroblasts were rinsed with PBS when cells became confluent and refreshed with DMEM supplemented with 10% FBS. After 24 h, the media was collected and precleaned by centrifugation. **b** Cortical tissues isolated from neonatal SD rats were used to prepare mixed glia. DMEM/F12 with 10% FBS was changed every 3 days. On the 9th day, mixed glia were cultured with media consisting of 75% DMEM/F12 (10% FBS) and 25% F-CM, defined as the F-CM group. Comparatively, the mixed glia cultured with media consisting of 75% DMEM/F12 (10% FBS) and 25% normal DMEM (10% FBS) were defined as the NM group. Microglia were isolated via mild trypsinization on the 13th day

Primary rat microglia cultures were prepared from the cerebral cortices of 1- to 2-day-old neonatal Sprague–Dawley rat pups, as previously described with some minor modifications (Tamashiro et al. 2012). Briefly, after removing the meninges, the cortices were dissected, cut into 2-mm pieces, and digested with 0.25% trypsin for 30 min at 37 °C, followed by suspension in DMEM/F12 with 10% FBS and 1% P/S and mechanical trituration with a P-1000 plastic tip. Then, the mixed cells were passed through a 70-μm nylon mesh cell strainer and plated on cell culture plates. The media were completely replaced every three days. When glial cells reached 70–90% confluency at approximately 9 days, the prepared F-CM were mixed with fresh glial cell culture media at a volume ratio of 1:3 (conditioned media: DMEM/F12 with 10% FBS) for further cell culture. In the non-treated control group, DMEM containing 10% FBS was used instead of F-CM. Approximately 13 days after the initial plating, microglia were isolated from mixed glial cultures via mild trypsinization according to the methods used in a previous study (Lin et al. 2017). Detachment of a layer of cells occurred when incubating mixed glial cultures with a trypsin solution (0.25% trypsin–EDTA 1:4 DMEM/F12) for 30 min. Microglial cells remained attached to the bottom of the well. The procedure of isolated microglia is illustrated in Fig. 1. To compare the yield, the cells from one brain of a neonatal pup were plated in one 6-well plate, and the cell density was calculated per field (five random fields of 100 × magnification per culture, *n* = 5 cultures). Only the first passage of the primary microglia were applied for the further studies. In addition, microglial cell cultured in 6-well plates were digested and reseeded for immunocytochemistry and phagocytic function assay.

### Immunocytochemistry

Microglial morphology was assessed by immunocytochemical detection of Iba-1 according to a previously published method (Hoogland et al. 2015). The cells were fixed in 4% paraformaldehyde for 30 min and washed three times with PBS, followed by permeabilization with 0.3% Triton X-100 in PBS for 15 min and blocking with 5% bovine serum albumin (BSA) for 1 h. Then, samples were incubated with a primary antibody against Iba-1 (1:500) at 4 °C overnight. After incubation, the cells were washed three times for 5 min with PBS and incubated with an Alexa Fluor 488-conjugated secondary antibody (1:800) for 1 h at room temperature. Then, the nuclei were stained with DAPI for 5 min. All stained samples were observed and imaged using a Nikon ECLIPSE 80i fluorescence microscope at ×100 or ×200 magnification.

### Flow Cytometry

The purity of microglia obtained from mixed glial cultures was estimated by flow cytometry. After mild trypsinization, the cells were collected and incubated with fluorochrome conjugated antibodies recognizing CD11b (1:20) and CD45 (1:20) at 4 °C for 20 min. Then, the cells were washed three times in cold PBS and resuspended at a final volume of 500 μl. Flow cytometry was performed on a BD FACSAria™, and data were analyzed using FlowJo software (Informer Technologies, USA).

### Microglial Phagocytic Function Assays

Phagocytosis is essential for microglial clearance of apoptotic cells, extracellular protein aggregates, and infectious bacteria in the CNS (Xing et al. 2014). Phagocytic function was assessed based on the uptake of fluorescein-labeled beads according to the methods of a previous study with some modifications (Lian et al. 2016). In brief, microglial cells cultured in 6-well plates were digested and reseeded in a 24-well plate coated with PDL at a concentration of 10^5^ cells/well. After 24 h, the cells were incubated with fluorescein-labeled beads at 37 °C for 2 h. To test the phagocytic function of microglia after stimulation, microglia were first stimulated with LPS (100 ng/ml), IL-4 (20 ng/ml), or IFN-γ (20 ng/ml) for 6 h, then the culture medium was discarded. Microglia was washed with PBS, and were incubated with fluorescein-labeled beads at 37 °C for 2 h. Iba-1 was used to reveal the cell shape as described above. Green fluorescent beads was visualized with the green channel and for Iba-1 staining, the red channel was used. Five random fields were captured for one culture plate, and each group included at least three culture plates. All images were captured with a fluorescence microscope and analyzed by a blinded observer with ImageJ software (Bethesda MD, USA).

### Real-Time PCR

Quantitative real-time PCR was used to measure mRNA expression. After mild trypsinization, microglia were cultured for 48 h with DMEM/F12 (10% FBS). Then, IL-4 (20 ng/ml), LPS (100 ng/ml), or IFN-γ (20 ng/ml) was used to treat cultured microglia for 8 h. Total RNA was extracted from primary cultured microglia using a miRNeasy kit (Qiagen, Hilden, Germany). One microgram of RNA was reverse-transcribed into cDNA using the PrimeScript™ RT Reagent Kit (Takara Bio, Tokyo, Japan). RT-PCR was performed using a quantitative PCR system (CFX Connect™ Real-Time System, Bio-Rad) with primers and a fluorescent dye (iQ™ SYBR® Green Supermix, Bio-Rad). Relative baseline genes (CD206, IGF-1, Arg1, IL-6, IL1-β, iNOS, TNF-α, and CD86) and microglial receptor genes (TLR2, TLR3, TLR4, P2Y12, P2Y6 and CX3CR1) levels were calculated by subtracting the Ct value of β-actin from the Ct value of the detected genes. Changes in mRNA expression (fold change) after various treatments were determined using the 2^−ΔΔCt^ method. All real-time PCR assays were performed in triplicate, and all experiments were independently repeated at least three times. Corresponding primers (Invitrogen, Thermo fisher scientific, USA) are listed in Table 1.

**Table 1:** Primers for RT-PCR

Genes		Primers (5′-3′)
β-actin	Forward	CACTGCAAACGGGGAAATGG
	Reverse	TGAGATGGACTGTCGGATGG
TNF-α	Forward	CAAGGGACAAGGCTGCCCCG
	Reverse	GCAGGGGCTCTTGACGGCAG
IL-6	Forward	AGAAGGAGTGGCTAAGGACCAA
	Reverse	AACGCACTAGGTTTGCCGAGTA
IL-1β	Forward	AAGCCTCGTGCTGTCGGACC
	Reverse	TGAGGCCCAAGGCCACAGGT
iNOS	Forward	CAGCTGGGCTGTACAAACCTT
	Reverse	CATTGGAAGTGAAGCGTTTCG
CD86	Forward	CCAGATTGCAGGTCCCAGTT
	Reverse	TCGACTCGTCAACACCACTG
IGF1	Forward	CTCCTCGCATCTCTTCTACC
	Reverse	GGACAGAGCGAGCTGACT
CD206	Forward	TTCGGTGGACTGTGGACGAGCA
	Reverse	ATAAGCCACCTGCCACTCCGGT
Arg1	Forward	TGCCCTCTGTCTTTTAGGGC
	Reverse	CCTCGAGGCTGTCCCTTAGA
TLR2	Forward	TGCTTTCCTGCTGAAGATTT
	Reverse	TGTACCGCAACAGCTTCAGG
TLR3	Forward	TCTGCACGAACCTGACAGAG
	Reverse	CAGTTGGACCCAAGTTCCCA
TLR4	Forward	CCGCTCTGGCATCATCTTCA
	Reverse	TGGGTTTTAGGCGCAGAGTT
P2Y6	Forward	ATGCCTGTTCACTGCCCCTA
	Reverse	CACAGCCAAGTAGGCTGTCT
P2Y12	Forward	GCACCAGATGCCAGTCTGCAAG
	Reverse	GGCACCTCCATGGTCCTGGTT
CX3CR1	Forward	GGACTGGGTGAGTGGCTGGC
	Reverse	GTGAGGTCCTGAGCAGCTGGG

### Statistical Analysis

All data are presented as the mean ± SD. All of the data were tested for normality and homogeneity of variance before analysis. Data of time-course experiments and real-time PCR for measurement of M1 (CD86, iNOS) or M2 (CD206, Arg1) markers were analyzed using two-way ANOVA. The mRNA expression levels of IL-6, TNF-α, IL1-β and IGF-1 after various treatments were analyzed using one-way ANOVA. Other data were analyzed using a *t* test. All experiments were independently conducted at least three times. Data were analyzed with GraphPad Prism 7.0 software (GraphPad, San Diego, CA, USA), and a value of *p* < 0.05 indicated statistical significance.

## Results

### Morphology and Yield

**Fig. 2:**
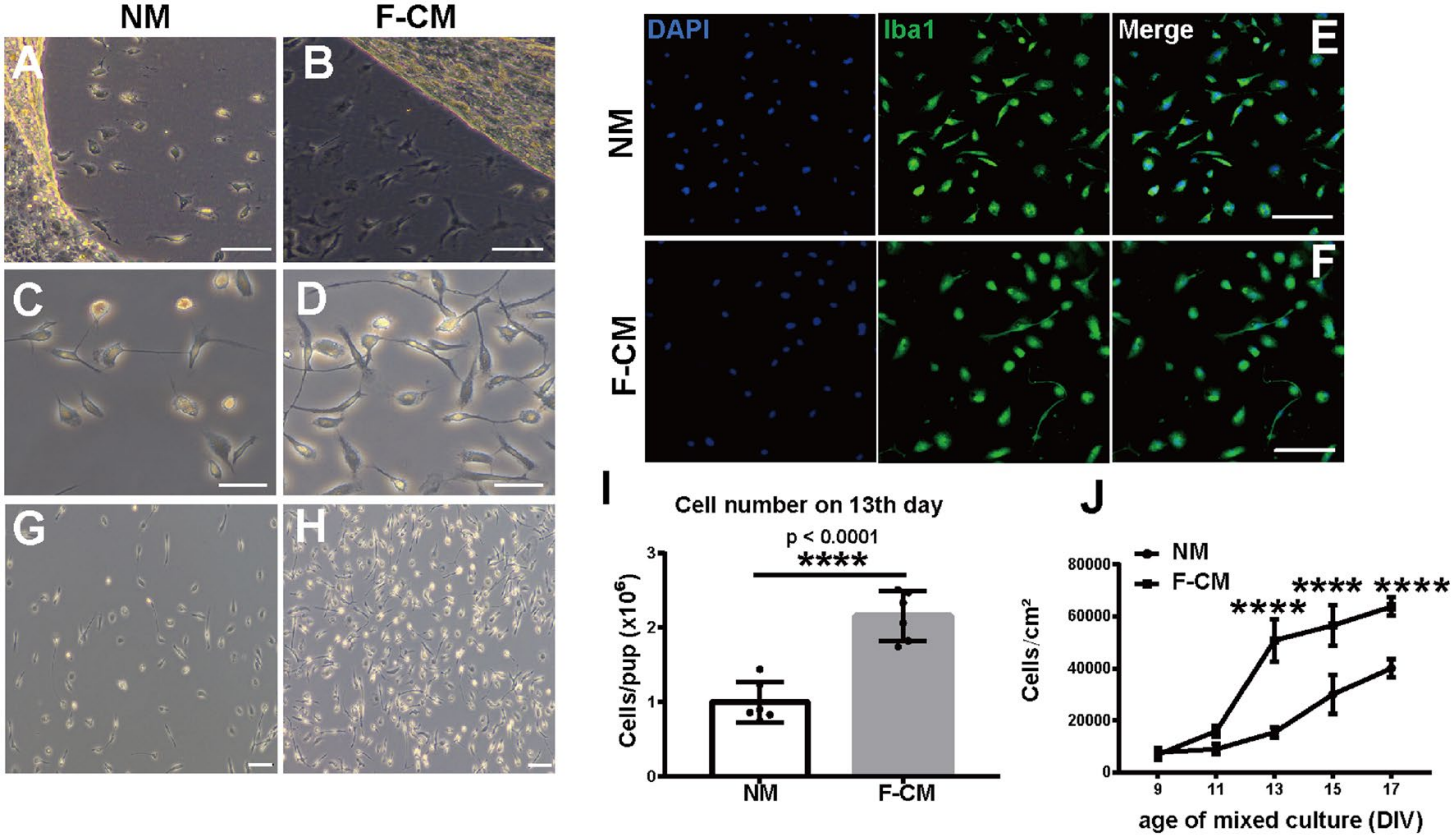
Morphology and yield of primary microglia obtained from the two groups. **a, b** show the progressive and rapid detachment of the mainly astrocytic layer of cells and the presence of a population of cells attached to the surface of the well with a microglial appearance in the NM (**a**) and F-CM (**b**) groups. Scale bar = 100 μm. **c**, **d** Morphology of microglia cultured for 24 h after isolation in the NM group (**c**) and F-CM group (**d**). Scale bar = 100 μm. **e**, **f** Iba-1 immunostaining of primary microglia isolated in the NM (**e**) and F-CM (**f**) groups. Scale bar = 100 μm. **g**, **h**, **i** The yield of microglia from the NM (**g**) and F-CM (**h**) groups. Scale bar = 50 μm. **j** Microglial cell density was affected by the age of the murine primary mixed glial culture. *n* = 6. ****p < 0.0001

Incubation of mixed glial cultures with a trypsin solution (0.25% trypsin–EDTA diluted 1:4 in DMEM-F12) after 30 min resulted in the detachment of an upper layer of cells in one piece, whereas a number of cells remained attached to the bottom of the well. After detachment of the main astrocytic layer of cells, cells with a microglial appearance were visible (Fig. 2a, b). After terminating trypsinization, the morphology of microglia isolated was observed by phase contrast microscopy. As the results shown, no significant differences were observed in the microglial morphology between the two groups, with both exhibiting a small cell body and ramified morphology (Fig. 2c, d). However, most microglia isolated from the F-CM group showed longer ramification (Fig. 2d). Iba-1 is typically used to distinguish microglia (Hoogland et al. 2015), and cells obtained by both methods were Iba-1 positive (Fig. 2e, f). On 13th day of culture, the yield of microglia from the F-CM group was 2.155 ± 0.1353 × 10^6^ cells/pup, which was approximately 2.1-fold higher than that of the NM group (1.002 ± 0.1107 × 10^6^ cells/pup) (*t* = 6.597, df = 10, *p* < 0.0001,* t* test) (Fig. 2g–i). We also performed a time-course experiment to reveal the changes in yield at different age of mixed culture indicated by days in vitro (DIV) in the two groups. The results showed that the yield of microglia in the NM group increased gradually, while a rapid increase occurred on the 13th day in the F-CM group. After 13 days, the change in yield in the F-CM group was gentle in late period (for days, *F*_4,50_ = 198.4, *p* < 0.0001; for methods, *F*_1,50_ = 224.2, *p* < 0.0001; and for methods*days, *F*_4,50_ = 29.52, *p* < 0.0001, two-way ANOVA) (Fig. 2j). In general, approximately 15 days were required to obtain a sufficient amount of microglia using F-CM to culture mixed glia, which was a shorter duration than when using the normal method.

### Size and Purity

**Fig. 3:**
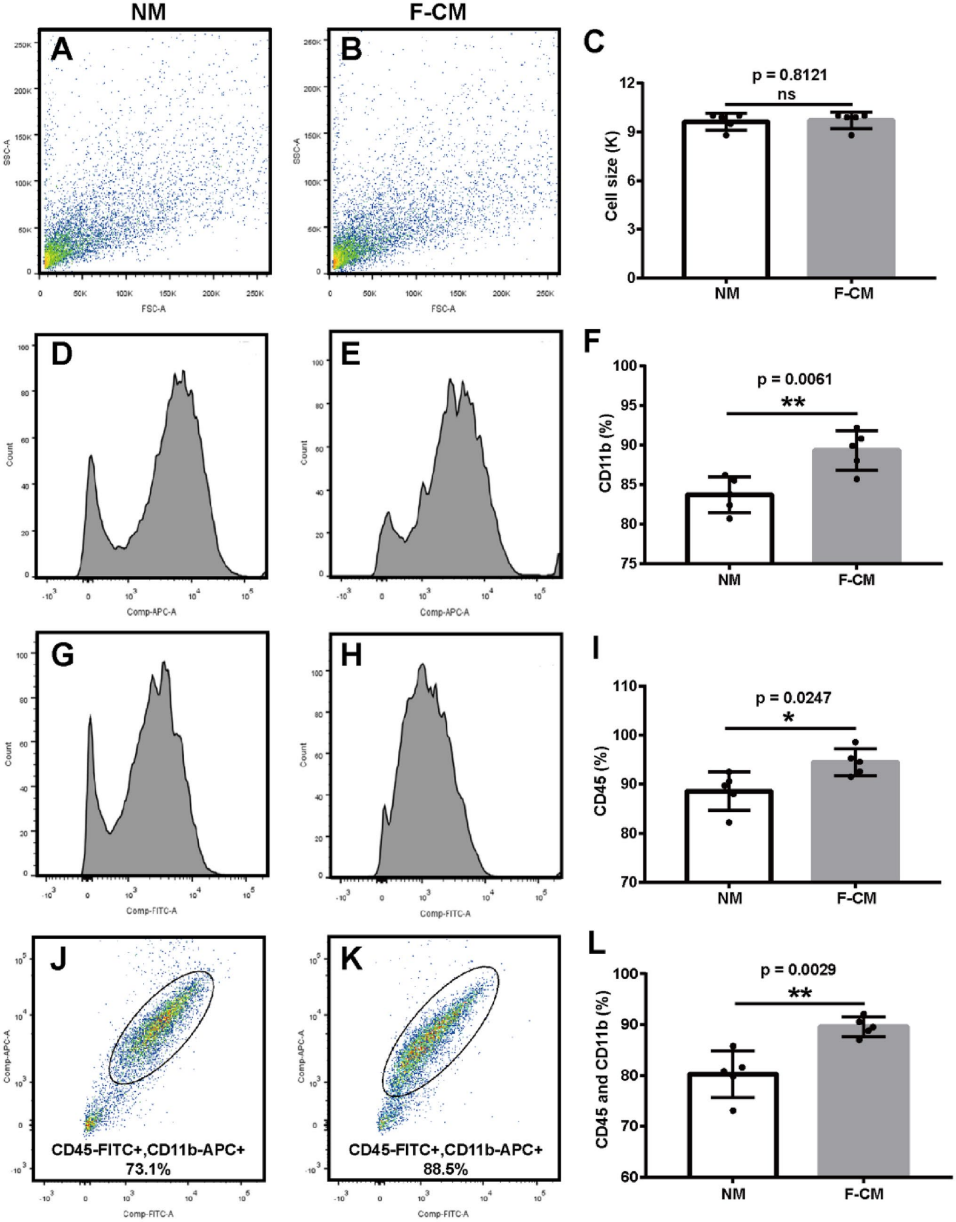
The size and purity of microglial cells obtained in the F-CM and NM groups. **a–c** The size of primary microglia isolated from the NM (**a**) or F-CM (**b**) groups. *n* = 3. **D**–**l** The purity of primary microglia isolated from the NM (**d, g, j**) or F-CM (**e, h, k**) groups was determined by flow cytometry with CD11b and CD45 staining. CD11b (**d**–**f**) and CD45 (**g–i**) were independently analyzed or determined together (**j–l**) using FlowJo software. *n* = 5. *p < 0.05; **p < 0.01; ns: no significant difference

Flow cytometry was performed to determine the size and purity of microglia. Flow cytometry with forward scatter (FSC) was measured, and the average sizes of microglia obtained from the NM group (9.567 ± 0.3844 K) and F-CM group (9.675 ± 0.2926 K) were similar (*p* = 0.5863, *t* test) (Fig. 3a–c). The purity of cultured microglia was analyzed by flow cytometry with CD11b and CD45 staining. The proportions of CD11b^+^ (Fig. 3d–f), CD45^+^ (Fig. 3g–i) and CD45^+^/CD11b^+^ (Fig. 3j–l) cells were all higher in the F-CM group (89.32 ± 1.133%, 94.52 ± 1.226%, and 89.58 ± 0.850%, respectively) than in the NM group (83.72 ± 1.005%, 88.60 ± 1.757%, and 80.22 ± 2.052%, respectively) (*t* = 3.698, df = 8, *p* = 0.0061; *t* = 2.763, df = 8, *p* = 0.0246; and *t* = 4.213, df = 8, *p* = 0.0029, for CD11b^+^, CD45^+^, and CD45^+^/CD11b^+^ cells, respectively, *t* test).

### Phagocytic Function and Baseline Gene Expression

**Fig. 4:**
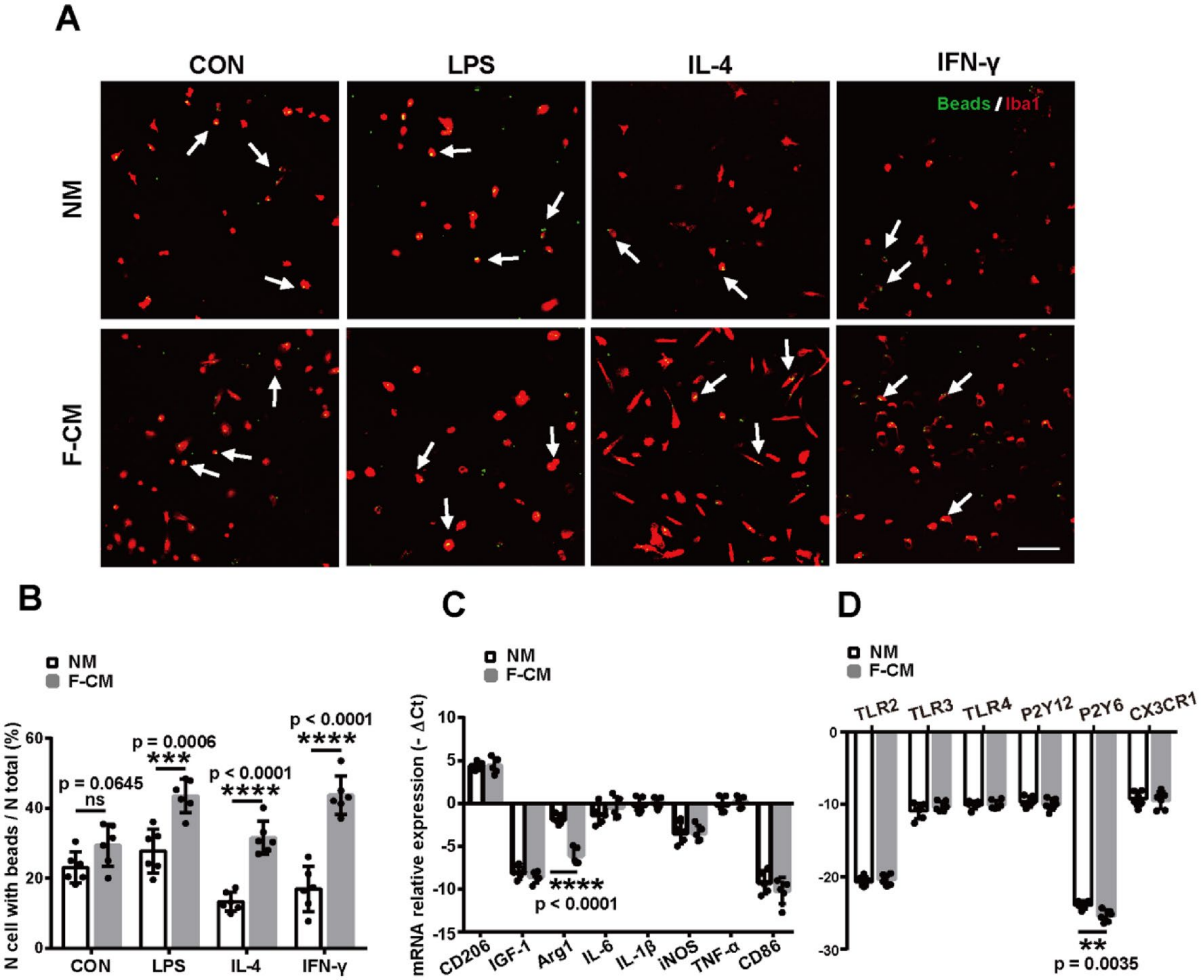
Phagocytosis and baseline levels of mRNA expression of primary cultured microglia isolated from the two groups. **a** Representative images of microglial (red) phagocytosed beads (green) after treatment with 100 ng/ml of LPS, 20 ng/ml IL-4 or 20 ng/ml of IFN-γ for 6 h. Scale bar = 100 μm. **b** The quantification of phagocytic ability. *n* = 6. **c** Baseline levels of gene expression. *n* = 5. **d** Baseline levels of microglial receptor mRNA expression. *n* = 6. **p < 0.01; ***p < 0.001; ****p < 0.0001; ns: no significant difference

Although the morphology of microglia appeared to be similar in the two groups, the properties of cells were still unknown. We next determined whether these cells might also demonstrate similar phenotypes. To assess the phagocytic activity of cells, we incubated microglia with fluorescent beads. The percentage of phagocytic cells was determined as: No. cell with beads/No. total cells. Some beads were captured by the microglia, and more obvious morphological changes were observed in F-CM group compared with NM group (Fig. 4a). The proportions of microglia containing beads in two groups were both approximately 25% (23.08 ± 1.817% for the NM group and 29.44 ± 2.465% for the F-CM group) (*t* = 2.007, df = 10, *p* = 0.0645, *t* test) (Fig. 4b), indicating that microglia obtained by the two methods possess similar phagocytic activity at baseline levels. However, the microglia in F-CM group showed stronger phagocytic ability than NM group after stimulated with LPS (27.77 ± 2.57% for the NM group and 43.6 ± 1.944% for the F-CM group, *t* = 4.911, df = 10, *p* = 0.0006, *t* test), IL-4 (13.35 ± 1.14% for the NM group and 31.56 ± 1.938% for the F-CM group, *t* = 8.101, df = 10, *p* < 0.0001, *t* test) and IFN-γ (16.95 ± 2.627% for the NM group and 43.75 ± 2.252% for the F-CM group, *t* = 7.747, df = 10, *p* < 0.0001, *t* test) ( Fig. 4b).

To assess various activation states of microglia, a representative panel of genes (CD206, IGF-1, Arg1, IL-6, IL1-β, iNOS, TNF-α, and CD86) was examined. The baseline expression level of these genes was similar, except for Arg1. The -ΔCt of Arg1 in the F-CM group (− 6.080 ± 0.4270) was smaller than that in the NM group (− 1.875 ± 0.2834), which suggested decreased expression of Arg1 mRNA in the F-CM group (*t* = 8.207, df = 8, *p* < 0.0001, *t* test) (Fig. 4c).

Phagocytosis is an important parameter to define the activation state of microglial cells. The activity of microglial phagocytosis relies on specific receptors expressed on the cell surface, such as Toll-like receptors (TLRs), pyrimidinergic P2 receptor subtypes, triggering receptor expressed on myeloid cells2 (TREM-2), scavenger receptors (SR) and so on, which contribute to the recognition and engulfment of harmful microparticles (Fu et al. 2014). Chemokine (C-X3-C motif) receptor 1 (CX3CR1) is known to affect the morphological/functional properties of microglia in the brain (de Cossío et al. 2021). Thus, some typical microglial receptor genes (TLR2, TLR3, TLR4, P2Y12, P2Y6, and CX3CR1) were also examined. The results showed that there was no significant difference in these receptor genes (TLR2, TLR3, TLR4, P2Y12, and CX3CR1), but the expression of P2Y6 mRNA in the F-CM group (− 25.06 ± 0.3246) was lower compared with the NM group (− 23.57 ± 0.2218) (*t* = 3.805, df = 10, *p* = 0.0035, *t* test) without external stimuli (Fig. 4d). That’s maybe the reason of no differences of microglial phagocytosis at the baseline level.

### Comparative Response to Stimulation

**Fig. 5:**
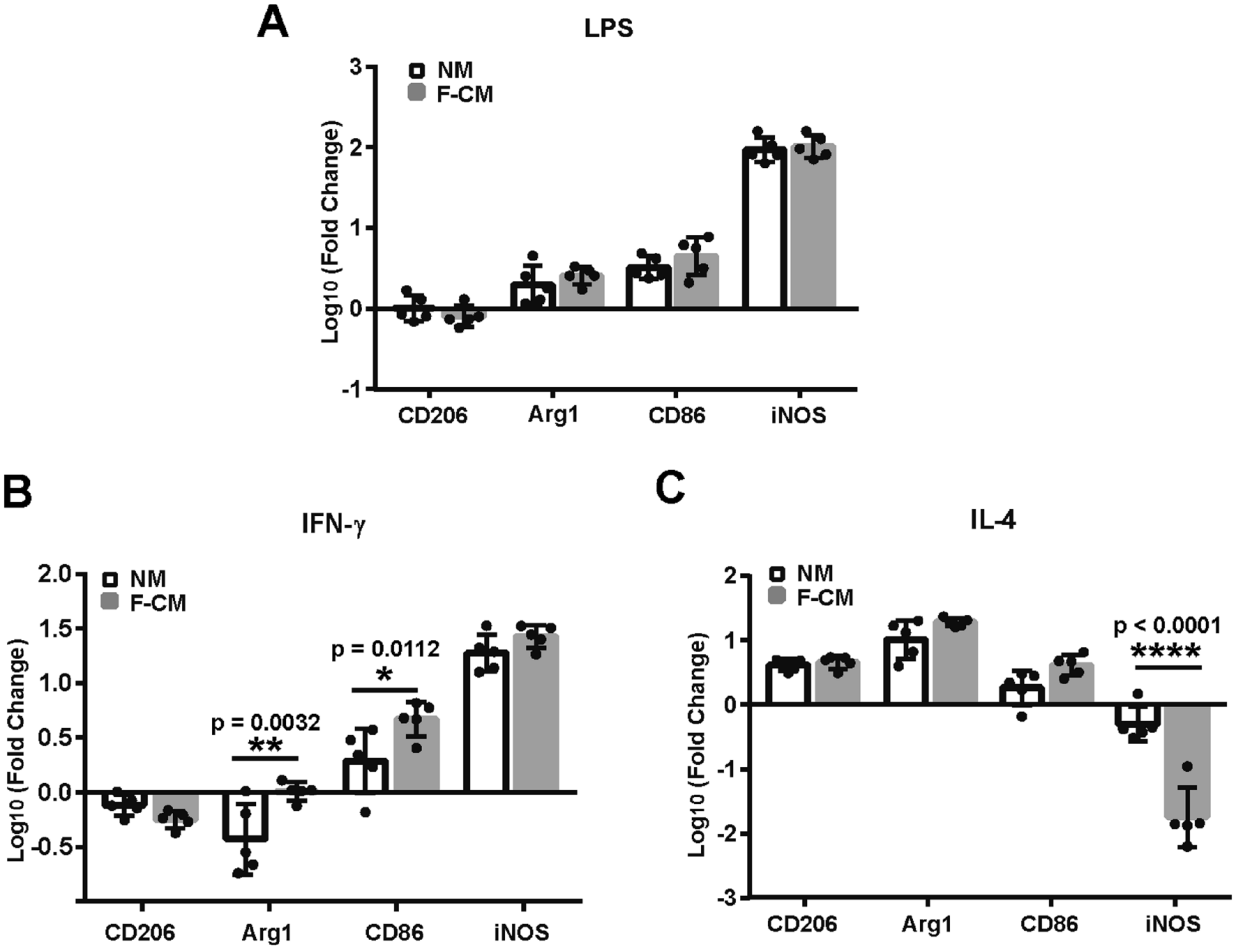
mRNA expression changes of M1 and M2 markers in primary cultured microglia isolated from the F-CM and NM groups after treatment with 100 ng/ml of LPS (**a**), 20 ng/ml of IFN-γ (**b**), or 100 ng/ml of IL-4 (**c**) for 8 h. *n* = 5. *p < 0.05; **p < 0.01; ****p < 0.0001

**Fig. 6:**
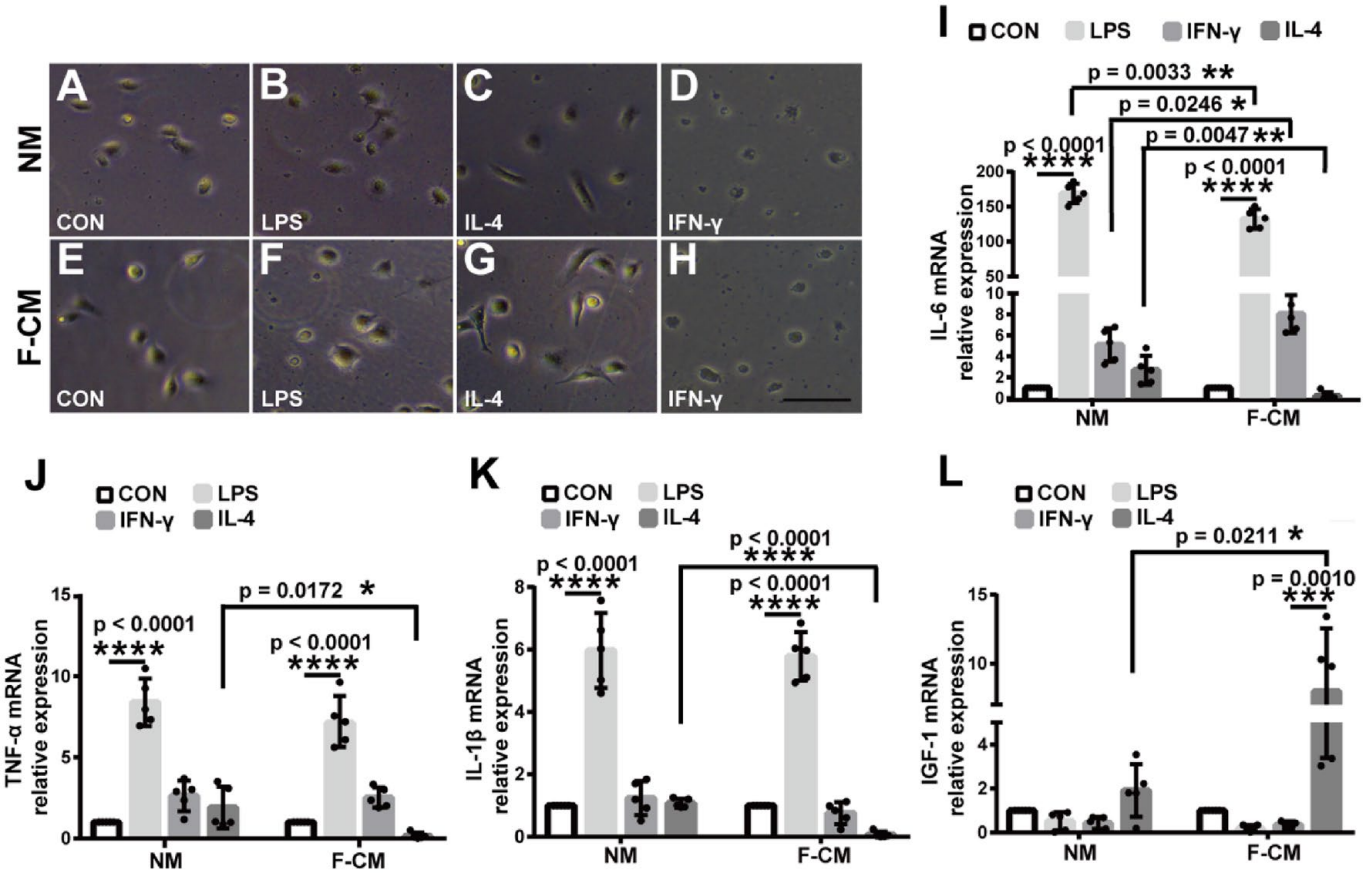
Morphological changes (**a–h**) and mRNA expression of cytokines (**i–l**) in primary microglia isolated from the F-CM or NM group after treatment with 100 ng/ml of LPS, 20 ng/ml of IFN-γ, or 100 ng/ml of IL4 for 8 h. Scale bar = 100 μm. *p < 0.05; **p < 0.01; ***p < 0.001; ****p < 0.0001

Next, we measured the response of microglia in the F-CM and NM groups to three types of stimuli (LPS, IFN-γ and IL-4) that are typically used to induce microglia into different phenotypes. LPS and IFN-γ can induce the M1 phenotype, while IL-4 mainly induces the M2 phenotype. After treatment with LPS (Fig. 5a), IFN-γ (Fig. 5b), and IL-4 (Fig. 5c) for 8 h, the mRNA expression of relevant M1 (CD86, iNOS) and M2 markers (CD206, Arg1) was assessed. LPS treatment upregulated expression of the M1 marker iNOS and downregulated expression of the M2 marker CD206 (for methods, *F*_1,32_ = 198.4, *p* = 0.3757; for genes, F_3,32_ = 276.5, *p* < 0.0001; and for methods*genes, *F*_3,32_ = 1.009, *p* = 0.4013, two-way ANOVA). The responses of microglia to LPS appeared to be generally similar CD206 (*t* = 0.935, DF = 32, *p* = 0.8288), Arg1 (*t* = 1.035, DF = 32, *p* = 0.7714), CD86 (*t* = 1.323, DF = 32, *p* = 0.5805), iNOS expression (*t* = 0.740, DF = 32, *p* = 0.9930, two-way ANOVA) (Fig. 5a). IFN-γ treatment decreased the expression of the M2 markers CD206 and Arg1 and increased the expression of the M1 markers iNOS and CD86 (for methods, *F*_1,32_ = 12.61, *p* = 0.0012; for genes, *F*_3,32_ = 155.6, *p* < 0.0001; and for methods*genes, *F*_3,32_ = 4.825, *p* = 0.007, two-way ANOVA). There was no significant difference for the responses to IFN-γ between NM and F-CM cultured microglia for CD206 (*t* = 1.125, DF = 32, *p* = 0.7147) and iNOS (*t* = 1.292, DF = 32, *p* = 0.6016). However, Agr1 expression (*t* = 3.697, DF = 32, *p* = 0.0032) and CD86 expression (*t* = 3.237, DF = 32, *p* = 0.0112, two-way ANOVA) presented significant difference (Fig. 5b). IL-4 increased the expression of Arg1, CD206, and CD86 and decreased the expression of iNOS in microglia from both groups (for methods, *F*_1,32_ = 6.223, *p* = 0.0180; for genes, *F*_3,32_ = 139.9, *p* < 0.0001; and for methods*genes, *F*_3,32_ = 28.59, *p* < 0.0001, two-way ANOVA). There was no significant difference for the responses to IL-4 between NM and F-CM cultured microglia for CD206 (*t* = 0.2465, DF = 32, *p* = 0.9986), Arg1 (*t* = 1.703, DF = 32, *p* = 0.3389), and CD86 (*t* = 2.230, DF = 32, *p* = 0.1252). while the downregulation of iNOS in the F-CM group was obviously attenuated compared to the NM group (*t* = 9.168, DF = 32, *p* < 0.0001, two-way ANOVA) (Fig. 5c). Although the gene expression varied, morphological changes appeared to be similar in the two groups after treatments for 8 h (100 ng/ml of LPS, 20 ng/ml of IFN-γ, or 20 ng/ml of IL-4) (Fig. 6a–h).

For further comparison, we determined the mRNA expression of inflammatory factors (IL-6, TNF-α, IL1-β) and a growth factor (IGF-1) after 8 h of treatment with the IL-4, LPS, or IFN-γ. Stimulation with IL-4, LPS, or IFN-γ induced distinct responses. We found that the microglia from NM and F-CM group had similar stimulus response by treating with LPS and IFN-γ. LPS significantly affected the expression of IL-6 (for NM group: *q* = 53.03, DF = 16, *p* < 0.0001; for F-CM group: *q* = 42.04, DF = 16, *p* < 0.0001), TNF-α (for NM group: *q* = 15.29, DF = 16, *p* < 0.0001; for F-CM group: *q* = 16.37, DF = 16, *p* < 0.0001), and IL1-β (for NM group: *q* = 16.76, DF = 16, *p* < 0.0001; for F-CM group: *q* = 24.91, DF = 16, *p* < 0.0001), while IGF-1 (for NM group: q = 1.663, DF = 16, *p* = 0.6498; for F-CM group: *q* = 0.7959, DF = 16, *p* = 0.9416, one-way ANOVA) had little effect (Fig. 6i-l). IFN-γ did not markedly affect the expression of IL-6 (for NM group: *q* = 1.293, DF = 16, *p* = 0.7975; for F-CM group: *q* = 2.254, DF = 16, *p* = 0.4095), TNF-α (for NM group: *q* = 3.367, DF = 16, *p* = 0.1213; for F-CM group: *q* = 4.019, DF = 16, *p* = 0.0519), IL-1β (for NM group: *q* = 0.7956, DF = 16, *p* = 0.9417; for F-CM group: *q* = 1.243, DF = 16, *p* = 0.8158), and IGF-1 (for NM group: *q* = 1.946, DF = 16, *p* = 0.5312; for F-CM group: *q* = 0.6282, DF = 16, *p* = 0.9698, one-way ANOVA) (Fig. 6i–l). Similarly, IL-4 did not obviously affect the expression of IL-6 (for NM group: *q* = 0.534, DF = 16, *p* = 0.9810; for F-CM group: *q* = 0.2553, DF = 16, *p* = 0.9978), TNF-α (for NM group: *q* = 1.866, DF = 16, *p* = 0.5644; for F-CM group: *q* = 2.220, DF = 16, *p* = 0.4220), and IL-1β (for NM group: *q* = 0.2608, DF = 16, *p* = 0.9972; for F-CM group: *q* = 4.841, DF = 16, *p* = 0.0596). However, it should be noted that IL-4 increased the expression level of IGF-1 in the F-CM group (*q* = 6.801, DF = 16, *p* = 0.0010) but not in the NM group (*q* = 3.154, DF = 16, *p* = 0.1574, one-way ANOVA) (Fig. 6i–l). Then, we further assessed the differences of the extent of responses. Compared to the baseline release level, LPS treatment induced significantly higher levels of IL-6 in microglia in the NM group (168.8 ± 6.257) than those in the F-CM group (132.4 ± 6.197) (*t* = 4.129, df = 8, *p* = 0.0033) (Fig. 6i), and other mRNA level (TNF-α, IL-1β, and IGF-1) had no difference (Fig. 6j–l). With IFN-γ treatment, both groups showed similar results (Fig. 6j–l), except for IL-6 level (5.092 ± 0.7052 vs. 8.045 ± 0.8041, *t* = 2.762, df = 8, *p* = 0.0246, *t* test). (Fig. 6i). The largest difference between the F-CM group and NM group was revealed after treatment with IL-4. As shown in Fig. 6i-l, IL-4 treatment significantly induced increased expression of IGF-1 in the F-CM group compared with CN group (7.979 ± 2.050 vs. 1.913 ± 0.5359, *t* = 2.863, df = 8, *p* = 0.0211, *t* test) but decreased the expression of IL-6 (0.2020 ± 0.1751 vs. 2.689 ± 0.6179, *t* = 3.873, df = 8, *p* = 0.0047, *t* test), TNF-α (0.1575 ± 0.09046 vs. 1.902 ± 0.5755, *t* = 2.995, df = 8, *p* = 0.0172, *t* test), and IL1-β (0.07092 ± 0.04418 vs. 1.077 ± 0.06357, *t* = 13.00, df = 8, *p* < 0.0001, *t* test) to less than baseline levels.

## Discussion

Microglia have multiple functions, especially in immune surveillance and inflammatory responses (Nimmerjahn et al. 2005; Hanisch and Kettenmann 2007; Zarruk et al. 2018). Microglial activation is critically involved in several pathological conditions such as age-related macular degeneration (AMD), Alzheimer’s disease, multiple sclerosis and stroke (Cai et al. 2014; Lee et al. 2014; Salter and Stevens 2017; Malpetti et al. 2020). Therefore, the ability to culture primary microglia in vitro will be an important tool in understanding their pathophysiology and functions. Although primary microglia are a better choice for experiments, many researchers still use the BV2 microglia cell line instead of primary microglia, mainly due to the low yield of microglia and the time-consuming procedure. Many protocols are available for culturing primary microglial cells from neonatal rodent brains, such as the classical shaking method (Giulian and Baker 1986). In 2003, mild trypsinization was introduced to obtain a higher yield of microglia (Saura et al. 2003). In 2017, our laboratory compared microglia isolated using the two methods and found that mild trypsinization generated a higher yield and purity than shaking, and microglia isolated by mild trypsinization appeared to be in a quiescent state with ramified morphology (Lin et al. 2017). However, the yield of microglia obtained by mild trypsinization still cannot meet the needs of some experiments such as western blots.

Shaking and mild trypsinization are different methods used to obtain microglia from mixed glia, but the procedure for culturing mixed glia is similar. Meninges and small blood vessels were dissected microscopically from brain tissue to reduce the contamination of mixed glial cells with vascular endothelial cells. Then, the cell suspension maintained in DMEM/F12 with 10% FBS and 1% penicillin–streptomycin (P/S), that procedure can inhibit neuron growth and grow a confluent mixed astrocyte/microglia population, consisted of approximately 35% neurons, 54% astrocytes, 9% microglia and a small percentage (< 1%) of other cells (He et al. 2018; Huang et al. 2015). The above processed are similar in both mild trypsinization and shaking method for microglia isolation. Mild trypsinization was first reported by Saura et al., they found that incubation of mixed glial cultures with 0.25% trypsin–EDTA diluted 1:4 in DMEM/F12 resulted in the detachment of an upper layer of cells in one piece, whereas a number of cells identified as > 98% microglia remained attached to the bottom of the well. Although the principle of mild trypsinization microglia isolation from mixed glial cells was not clear, Saura et al. pointed that the presence of Ca^2+^ is necessary for the trypsin-induced separation. Since the Ca^2+^ concentration was higher than the chelating capacity of the EDTA present, the non-sequestered free Ca^2+^ partially inhibited trypsin and this induced the observed partial trypsinization, resulted in the detachment of an intact layer of cells containing virtually all the astrocytes and other cells, leaving a population of firmly attached microglia (Saura et al. 2003). In addition, mild trypsinization process resembles an accelerated form of that occurring when confluent mixed glial cultures are nutritionally deprived, which gives rise to a progressive retraction of astrocytes, and results in the appearance of microglial cells attached to the bottom of the well (Hao et al. 1991).

Previous study reported medium was additionally supplemented with 30% mouse fibroblast cell line L929-conditioned DMEM after establishment of an astrocytic monolayer to isolate primary microglia from Alzheimer’s disease model *APPPS1* and wildtype mice (Krabbe et al. 2013). Although Krabble et al. (2013) suggested mouse fibroblast cell line can secret CSF to stimulate microglial proliferation, there is no further information about the comparison of microglia from normal media and fibroblast-conditioned media. In our study, we used mixed media consisting of 25% F-CM and 75% DMEM/F12 (10% FBS) to culture mixed glia and found that the yield of microglia isolated from mixed glia treated with F-CM was significantly increased (see Fig. 2g–i), and the purity was higher (see Fig. 3d–l). The culture time required to obtain a high yield of microglia was approximately 13 days (see Fig. 2j), which is a shorter time than needed when using the normal culture method (approximately 20 days) (Saura et al. 2003). Microglia are highly sensitive and reactive cells, it is possible that different methods may result in different phenotypes of microglial cells, which in turn can potentially influence experimental results. We compared the state of microglia according to three aspects: morphology, phagocytic function and baseline mRNA expression. The microglia isolated from the F-CM group had longer ramifications (Fig. 2c, d). In addition to some receptors on the microglia, it has been reported that microglial phagocytosis could be regulated by pro-and anti-inflammatory cytokines (Lively and Schlichter 2018; Koenigsknecht-Talboo and Landreth 2005), while the exact mechanism and modulation of microglial phagocytosis needs further study. In our study, microglia isolated from the F-CM group showed stronger phagocytic ability compared with NM group, after stimulating with LPS, IL-4 and IFN-γ (see Fig. 4a, b). We hypothesized that was due to the primary microglia from F-CM group were more sensitive to external stimuli, caused microglial more activated and higher phagocytosis. Overall, no significant difference was observed in the general state of microglia isolated from the F-CM and NM groups. Relative to “resting” microglia, activated microglia receive more attention. Microglial activation is not univalent or bivalent. The concept of classically activated (M1-like) and alternatively activated (M2-like) microglia is derived from studies of macrophages, but microglia are different from macrophages that reside in other tissues due to their cell-specific gene expression and differential functions (Ginhoux and Merad 2011; Ginhoux et al. 2010). Activated microglia present a wide spectrum of phenotypic and functional diversity, depending on the stimulus and context. M1- and M2-like microglia are widely investigated, and the so-called M1- or M2-like markers are also not always absolutely limited to one microglial phenotype (Tang and Le 2016; Miao et al. 2018). In our study, we examined changes in mRNA expression (iNOS, CD86, CD206, Arg1, IL-6, TNF-α, IL1-β, and IGF-1) in microglia obtained from these two groups after treatment with typical stimuli that may induce M1-like (LPS or IFN-γ) or M2-like (IL-4) phenotypes. As expected, the microglia obtained from either the F-CM group or NM group were functional and generally responded to different treatments. However, the extent of responses appeared to differ under some conditions. For example, the expression level of iNOS in microglia isolated from the F-CM group was reduced to a greater extent by IL-4 than in the NM group (Fig. 5c). A difference was also noted in the expression of IL-6, TNF-α, IL1-β, and IGF-1 when microglia were treated with IL-4 (Fig. 6i–l). The results suggested that microglia in the F-CM group were more sensitive to IL-4 treatment. Therefore, diversification of methods should be considered because even though both methods are feasible for evaluating the functional responses of microglia in vitro, opposite conclusions may arise if the extent of response is critical for a particular study.

Treating the mixed glial cultures with fibroblast-conditioned media, prior to microglia isolation, significantly increased the yield and purity of the obtained microglia, and markedly shortened the time length of the microglia-obtaining procedure, compared with the microglia treated with normal media. This could be explained by fibroblast-secreted factors in the F-CM might be responsible for the increases in microglial growth. Fibroblasts secrete various factors such as extracellular matrix proteins (e.g., collagen I and decorin), cytokines (e.g. IL-1, IL-6 and TNF-α), VEGF and diverse fibroblast growth factors (FGFs) (Murase et al. 1992; Costa et al. 2015; Jeong et al. 2016a; Russo et al. 2021). Collagen I and decorin were reported whose activity is required for controlling cell adhesion and migration (Lopatina et al. 2020). Some pro-inflammatory cytokines are able to upregulate microglial proliferation (Ganter et al. 1992). It has been reported that in fibrosis or tumors, fibroblasts are the main source of IL-6 and have influence on other cells through IL-6 in a paracrine manner (Shimodaira et al. 2018; Sundararaj et al. 2009). Costa et al (2015) reported that mouse embryonic fibroblasts (mEFs) can produce a amount of TNF-α. TNF increased microglial proliferation in mixed astrocyte-microglial cultures but had no mitogenic effects on isolated microglia (Ganter et al. 1992). Moreover, VEGF and FGF can promote a significant increase in cell proliferation (Pedersen et al. 2009).

Besides, fibroblast was reported to produce osteopontin (OPN), that is a phosphorylated acidic glycoprotein N contributes to fibrogenesis in the lung, liver, or heart (Shimodaira et al. 2018). OPN excessively enhances the migration and invasion of cells and induces extracellular matrix (Pedersen et al. 2009; Tambuyzer et al. 2012). Cytokine macrophage colony-stimulating factor (CSF), produced by activated astrocytes, macrophages and microglia, inflammatory cells, as well as fibroblast, is an important growth factor for microglia and macrophages (Raju et al. 2015; Krabbe et al. 2013). CSF is mitogens for microglia, maintenance of the microglia was improved by addition of the CSF (Raju et al. 2015). Previous studies showed microglial survival requires various factors such as TGF-β, and cholesterol (Butovsky et al. 2014; Bohlen et al. 2017). According to the above information, we speculate some cytokines and growth factors from F-CM and produced from the F-CM-stimulated astrocytes are possible candidate factors that affect proliferate, growth and maintenance of microglia.

This study has a few caveats. First, the proportion of F-CM in the mixed media we chose was 25%. We used different percentages, ranging from 10 to 40%, to culture cells in typical experiments and found that the effect on the yield depended on the percentage of F-CM. However, frequent media changes would be required if a high percentage of F-CM was used. The time when conditioned media was added depended on when astrocytes reached confluence because microglia began a phase of rapid proliferation at this time (Saura 2007). Second, we did not examine why F-CM improved the yield of microglia in this study. Third, although we showed that F-CM could improve the yield of microglia isolated using mild trypsinization, we do not know whether F-CM plays the same role when the shaking method is used to isolate microglia. Mild trypsinization mainly isolates the microglial cells that are located below the astroglial monolayer or intermingled among the astrocytes, and the microglia located on top of the astroglial monolayer could be recovered by shaking (Saura 2007). The possibility that F-CM only increases the yield of microglia under the astroglial monolayer cannot be excluded.

Culturing primary microglia is essential for many laboratories. Recently, modified or new methods have been used to efficiently obtain microglia, including the magnetic microbead method (Ju et al. 2015); and these methods are focused on the isolation step. We used fibroblast media to alter the method of culturing mixed glia, which is less expensive (no need for extra reagents and equipment) and more efficient (higher yield and shorter time).

## Conclusions

Our study demonstrates that a higher yield of microglia can be obtained in vitro from mixed glia cultured with F-CM than with the common culturing method. Additionally, the time required for the entire process is only approximately 13 days, and the microglia obtained exhibit no major differences. In summary, using F-CM will improve in vitro culture of microglia and facilitate investigations of the mechanisms of CNS diseases.
